# Population pharmacokinetics of teicoplanin and dosage optimization in sepsis patients based on continuous renal replacement therapy

**DOI:** 10.3389/fphar.2025.1621959

**Published:** 2025-07-01

**Authors:** Qian Sun, Jiang Jian, Xueqiang Zhou, Zhu Hong, Suwen Yang, Yanping Zheng, Siyi Wang, Maojun Zhao

**Affiliations:** ^1^ Department of Intensive Care Unit, Beijing Jishuitan Hospital Guizhou Hospital, Guiyang, Guizhou, China; ^2^ Department of Pathogenic microorganism, Bijie Medical College, Bijie, Guizhou, China; ^3^ Department of Clinical Laboratory Medicine, Kaiyang County People’s Hospital, Guiyang, Guizhou, China; ^4^ Department of Pharmacy, Huadu District People’s Hospital of Guangzhou, Guangzhou, China

**Keywords:** teicoplanin, pharmacokinetics, dosage optimization, sepsis, continuous renal replacement therapy

## Abstract

**Purpose:**

It is well known that pharmacokinetics (PK) of drugs is significantly altered in sepsis patients receiving continuous renal replacement therapy (CRRT). However, clinical studies investigating the PK of drugs administered during CRRT are limited, and appropriate dosing regimens have not yet to be definitively established. The study aimed to develop a population PK model for teicoplanin, explore significant covariates regarding to teicoplanin PK, and propose optimal dosage strategies for sepsis patients.

**Methods:**

Eighty-six sepsis patients were included and plasma samples from all patients were analyzed. PK analysis was conducted on samples from 86 sepsis patients, followed by population PK analysis and simulations to ascertain the probability of target attainment (PTA).

**Results:**

Teicoplanin was well characterized by a one-compartment PK model with first-order elimination. The presence of CRRT was associated with a lower volume of distribution (V), and gender was associated with a higher V. When MIC was set at 1 mg/L, a loading dose of 800 mg (q12h) followed by a maintenance dose of 600 mg (q24h) was necessary for male sepsis patients without CRRT, and a loading dose of 800 mg (q12h) followed by a maintenance dose of 800 mg (q24h) for male sepsis patients receiving CRRT. Female patients with sepsis required a loading dose of 1,000 mg q12h followed by a maintenance dose of 1,000 mg q24h.

**Conclusion:**

Teicoplanin therapy in sepsis patients undergoing CRRT necessitates individualized dosing. A PK model-based teicoplanin dosing regimen for sepsis patients with CRRT was proposed, whereas prospective clinical study is required to validate.

## 1 Introduction

Sepsis is defined as a life-threatening failure of vital organ function caused by a host’s dysfunctional response to infection (Cecconi et al., 2018; Li et al., 2023), with a prevalence of nearly 50 million annually worldwide and an all-cause mortality rate of 12.5%–31.8% (Li et al., 2023). Any infected person can potentially develop sepsis, and the incidence of sepsis is as high as 30% of intensive care unit (ICU) patients (Sakr et al., 2018). Teicoplanin, a glycopeptide antibiotic agent, exerts an excellent antibacterial activity against Gram-positive infection through inhibiting the cell-wall peptidoglycan systhesis of bacteria (Jung et al., 2009). It has been widely used for clinical application in life-threatening infections for its excellent antimicrobial effect against drug-resistant Gram-positive bacteria, especially methicillin-resistant *Staphylococcus aureus* (MRSA) (Yamaguchi et al., 2023; David and Daum, 2010; Kang et al., 2023) which is one of the main pathogenic bacteria in sepsis (David and Daum, 2010).

Teicoplanin has a relatively long elimination half-life of 30–180 h after administration and there is high protein binding (over 90%) to plasma albumin (Zhou et al., 2019; Boztug et al., 2017; Yamaguchi et al., 2023), which results in a great inter-individual variability (Yamaguchi et al., 2023). Therapeutic drug monitoring (TDM) has been shown to be clinically beneficial for teicoplanin in critical ill patients (Abdul-Aziz et al., 2020). Adequate exposure to ensure antimicrobial effect is confirmed as a minimum plasma concentration (Cmin) of >10 μg/mL for most Gram-positive bacteria, when measured by high-performance liquid chromatography (HPLC) (Abdul-Aziz et al., 2020; Wi et al., 2017). However, data demonstrated that about 48%–89% of patients failing to achieve the target therapeutic range when prescribed the standard dosage regimens (Zhang et al., 2020; Zhao et al., 2015). Also, 14.1% of Cmin was still lower than expected (<10 μg/mL) even though higher doses were prescribed for patients (Strenger et al., 2013), and the overall mean Cmin was 9.0 μg/mL (Lukas et al., 2004).

Notably, patients in ICU have marked homoeostatic change, driven by both the interventions required (i.g., CRRT) and the underlying disease process (i.g., systemic inflammatory, multiple organ dysfunction) (Abdul-Aziz et al., 2020). Critical illness like the sepsis is accompanied by an increase in capillary permeability to proteins, which can lead to the loss of protein-rich fluid from the intravascular to the interstitial space (defined as capillary leak syndrome). This syndrome can change the volume of distribution (V) for hydrophilic drugs (Gatti and Pea, 2021; Goulenok and Fantin, 2013), which results in reduced drug concentrations, and thereby hindering the attainment of therapeutic targets for time-dependent antibiotics (Goulenok and Fantin, 2013). Additionally, patients with sepsis -associated acute kidney injury required CRRT (Martensson and Bellomo, 2015). It can eliminate both of exogenous antibiotics and endogenous toxins, and this contributes to extracorporeal drug clearance and further lowers the drug concentrations. Therefore, the combined effects of critical illness and CRRT could significantly impact teicoplanin pharmacokinetics (PK) (Abdul-Aziz et al., 2020; Hoff et al., 2020). It means that developing an effective and safe of teicoplanin dosage regimen remains challenging in clinical practice in sepsis patients (Ueda et al., 2022; Hanai et al., 2022), particularly in those receiving CRRT.

Currently, limited relevant PK studies are available in sepsis patients undergoing CRRT, and no clear guidelines regarding proper dose recommendation in the setting of CRRT exist. The study aimed to describe the PK of teicoplanin in sepsis patients with/without CRRT, to characterize and quantify the factors contributing PK variability, and to propose optimal dosing regimens to ensure sufficient teicoplanin exposure.

## 2 Patients and methods

### 2.1 Study patients

This study was a retrospective PK study performed in the intensive care unit in Beijing Jishuitan Hospital Guizhou Hospital between 1 June 2022 and 1 June 2024. The study protocol was approved by the institutional review board of Beijing Jishuitan Hospital Guizhou Hospital (IRB No. KT2022102101), and conducted based on the principles of the current Good Clinical Practice and Declaration of Helsinki. Written informed consent was collected from the legal representatives of each patient.

The eligible criteria for patient inclusion in the study were: (1) patients aged ≥18 years old; (2) patients for proven sepsis according to Sepsis 3.0 criteria; (3) patients with confirmed or suspected Gram-positive bacteria; (4) patients receiving tecoplanin ≥4 days. Children, pregnant women, patients with infections in special conditions (joints, bone, endocardium, etc.), patients without a complete teicoplanin dosing history, or those who did not receive therapeutic drug monitoring were excluded.

Data for pharmacokinetic modeling were collected from the Medical system record. For each patient, the following information were included, but not limited to, demographic characteristics (age, gender and weight), physiological and biochemical parameters [white blood count (WBC), albumin (ALB), total protein (TP), aspartate transaminase (AST), alanine aminotransferase (ALT), total bilirubin (TBIL), direct bilirubin (DBIL), serum creatinine (Scr)], disease state (diagnosis, combined disease),special treatments [extracorporeal membrane oxygenation (ECMO), CRRT], medication information [dose, dosing interval, administration/sampling time, administration rate, comedication] and monitoring concentrations of teicoplanin.

### 2.2 Dosage regimens and pharmacokinetic sampling

Teicoplanin was administered through intravenous bolus infusion of 400 mg, q12h for the first three doses followed by the daily maintenance dose of 400 mg. The infusion duration was 1 hour. Therapeutic drug monitoring (TDM) was typically performed within 30 min preceding a dose at steady state. Blood samples were centrifuged for 3,000 r.p.m/min ×10 min at 4°C, and separated and stored at −80°C until analysis.

### 2.3 Teicoplanin assay

Teicoplanin concentrations were determined following protein precipitation using a validated high-performance liquid chromatography (HPLC) system with UV detection (Waters Inc., Milford, United States). Chromatographic separation was performed on a Hypersil C18 column (250 mm × 4.6 mm, particle size, 5 μm; Thermo Fisher Scientific Inc., Boston,United States) with a mobile phase consisting of 0.01 mol/L NaH_2_PO_4_: acetonitrile: methanol (70:25:5, vol/vol) at a flow rate of 1.0 mL/min. Plasma samples (400 μL) were mixed with 50 μL piperacillin sodium (internal standard, 0.15 mg/mL) and 600 μL of acetonitrile. After 2-min vortex mixing and 10-min centrifugation, the 600 μL of supernatant was transferred to a tube, and diluted with 400 μL of dichloromethane. Then the proceed sample were placed in an autosampler vials. A volume of 20 μL was injected into the HPLC system for analysis. The UV detection wavelength was set at 220 nm. The calibration curves proved acceptable linearity in the range of 5.63–125.00 mg/L (*r* ^2^ > 0.99). The accuracy and precision of quality control samples (7.81, 31.25, and 90.00 mg/L) ranged from 2.98% to 10.36, and 7.33%–11.25%, respectively. Results from validation of teicoplanin assay showed satisfaction in linearity, extraction efficiency and matrix effects.

### 2.4 Population pharmacokinetic modeling

Population pharmacokinetic (popPK) modeling was performed using Phoenix NLME™ (version 8.1; Certara L.P Pharsight, MO, United States). Graphical analysis was carried out with GraphPad Prism (version 8.0.2 Windows version, GraphPad Software, San Diego). The concentration-time data was analyzed using the first-order conditional estimation with interaction (FOCE-I) within a nonlinear mixed-effects framework. Initial PK analyses tested both one- and two-compartment models. Between-subject variability (BSV) for pharmacokinetic parameters was determined using an additive error model, as represented by the following equation (Equation 1):

$$P_{j}=\text{tv}\left(P\right)+\eta_{j}^{P},$$ (1)

where tv(P) denotes the typical population estimates of the pharmacokinetic parameters, P_j_ indicates the pharmacokinetic parameter of the jth individual, and η_j_^P^ represents the inter-individual variability. η_j_ follows a normal distribution around 0 with the variance of ω^2^. Various error models, including power, mixed (additive and proportional) and proportional were evaluated to describe the residual error.

### 2.5 Covariate model

Demographic and clinical characteristics that were considered plausible for affecting teicoplanin PK were evaluated as covariates. The potential covariates included gender, body weight, age, Scr, ALB, the CRRT status, etc. After obtained the initial PK estimates from the structural model, the covariate model was assessed by comparing the changes of the objective function value (OFV) and the diagnostic plots. The covariate was tested using a stepwise method with a forward-inclusion process and a backward-exclusion process. In the forward selection phase, a covariate was retained in the model if it resulted in a significant reduction (p < 0.05, decrease >3.84) in the OFV compared to the structural model. The significance of each variable was subsequently re-assessed through backward selection, where an increase in OFV exceeding 6.63 (p < 0.01) was necessary for confirmation.

Continuous covariates were tested by the power equation (Equation 2):

$$\text{tv}\left(P^{\prime }\right)=\text{tv}\left(P\right)\cdot {\left[{\text{Cov}}_{\text{con}}/\text{median}\left({\text{Cov}}_{\text{con}}\right)\right]}^{\theta_{\text{cov}}},$$ (2)

where θ_cov_ denotes the estimated coefficient of the covariate, and the continuous covariate (Cov_con_) was normalized by its median value.

Categorical covariates were tested by the following equation (Equation 3):

$$\text{tv}\left(P^{\prime }\right)=\text{tv}\left(P\right)\cdot \mathrm{EXP}\left(\theta_{\text{cov}}\cdot {\text{Cov}}_{\text{cat}}\right),$$ (3)

where Cov_cat_ is set to 0 or 1 for categorical covariates.

### 2.6 Model evaluation

The performance of the final model was evaluated by internal validations, including goodness-of-fit (GOF), bootstrap and prediction corrected visual predictive check (pc-VPC). GOF evaluation was performed by plotting the corresponding individual (IPRED) and population predictive values (PRED) against the observed values as well as the PRED and time against conditional weighted residual errors (CWRES). A bootstrap resampling technique was used for model validation. One thousand bootstrap-resampled data sets were generated from the original model group data set, and each was individually fitted to the final model. All parameters were estimated, and the median and 95% confidence intervals (CIs) (2.5th percentile and 97.5th percentile) were compared with the final parameter estimates. Pc-VPC was used to graphically assess the appropriateness of the compartment model based on 1,000 replicates of the dataset.

### 2.7 Monte Carlo simulations and dosing optimization

Monte Carlo simulations (n = 1,000) were performed using the final population PK model to evaluate the probability of target attainment (PTA) under various dosing regimens. The primary pharmacodynamic (PD) targets included a trough concentration (Cmin) ≥10 mg/L, which is recommended for the treatment of Gram-positive infections such as MRSA and is widely used in TDM of teicoplanin (Ariano et al., 2013; Liu et al., 2011). Additionally, an AUC/MIC ratio ≥345 was adopted as a secondary PD target, based on previous studies associating this threshold with optimal clinical outcomes (Matsumoto et al., 2016; Matsumoto et al., 2014). An MIC of 1 mg/L was assumed in accordance with EUCAST breakpoints for teicoplanin-susceptible *S. aureus*.

Teicoplanin was administrated at loading doses ranging from 600 mg to 1,200 mg every 12 h for either 3 or 5 doses, with maintenance doses ranging from 200 mg to 1,000 mg every day. Continue dosing regimens that administrated 400–1,000 mg every 12 h or 1,000–1800 mg every day were also investigated. For each medication scenario, 1,000 replications were performed. Trough concentrations on day 4 (C_72h_) and at steady state of day 7 (C_168h_) after initial dosing, as well as the relevant area under the curve from 48 to 72 h (AUC_48–72h_) and from 144 to 168 h (AUC_144–168h_) were simulated. The probability of target attainment (PTA) was calculated as the proportion of simulated patients achieving the target C_min_ or AUC in percentage.

## 3 Results

### 3.1 Patients’ characteristics

Demographic and clinical characteristics for each patient were shown in Table 1. In all, 86 patients (median (IQR) age 62.00 (53.00,71.25), 51 males and 35 females) met the inclusion/exclusion criteria. Twenty patients required CRRT, among of which one patient underwent both continuous venous hemofiltration (CVVH) and continuous venous hemodiafiltration (CVVHD), 5 patients underwent CVVHD only, and the remaining 14 patients received CVVH only. Four patients received extracorporeal membrane oxygenation (ECMO) treatment in ICU. A total number of observed teicoplanin concentrations (n = 86) for patients were collected. The median (IQR) trough concentrations were 13.40 (10.48,19.83) mg/L. Patients received 200–800 mg of teicoplanin dosing [median (IQR):400 (400,400)] as an initial the loading dose, followed by daily maintenance dose of 400 mg [median (range):400 (200, 1,000)].

**TABLE 1:** Characteristics of the patients.

Characteristics	Sepsis patients (n = 86)
Gender	
Males, n (%)	51 (59.30%)
Females, n (%)	35 (40.70%)
Age (years)	62.00 (53.00,71.25)
Weight (kg)	62.00 (51.88,70.00)
Height (cm)	165.00 (156.30,172.00)
Serum albumin concentration (mg/L)	31.60 (29.48,36.90)
Serum creatinine concentration (mg/dL)	109.00 (74.00,184.80)
Initial teicoplanin dose	400.00 (400.00,400.00)
Maintenance teicoplanin dose	400.00 (400.00,400.00)
Teicoplanin concentration (mg/L)	13.40 (10.48,19.83)
ECMO, n (%)	4 (4.65%)
CRRT, n (%)	20 (23.26%)

### 3.2 Population pharmacokinetic modeling

The PK profile of teicoplanin was effectively characterized by a one-compartment model with first-order elimination, incorporating with an additive residual variability as well as interindividual variability in the volume of distribution (V) and clearance (CL). Among the covariates screening proceed, the inclusion of gender and CRRT in the volume of distribution using a proportional model significantly enhanced the model fit, as evidenced by changes in objective function value (ΔOFV) = -6.51 and −17.82, respectively. No covariate was found to significantly influence the CL of teicoplanin. The final model was described by the Equations 4, 5.

$$\text{CL}\left(L/h\right)=0.98\times \mathrm{EXP}\left(0.31\right),$$ (4)

$$V\left(L\right)=108.69\times \mathrm{EXP}\left(-0.71\times \left(\text{if with CRRT}\right)\right)\times \mathrm{EXP}\left(-1.07\times \left(\text{if is Female}\right)\right)\times \mathrm{EXP}\left(0.09\right),$$ (5)

The typical population estimates for CL and V derived from the final model were.

**FIGURE 1:**
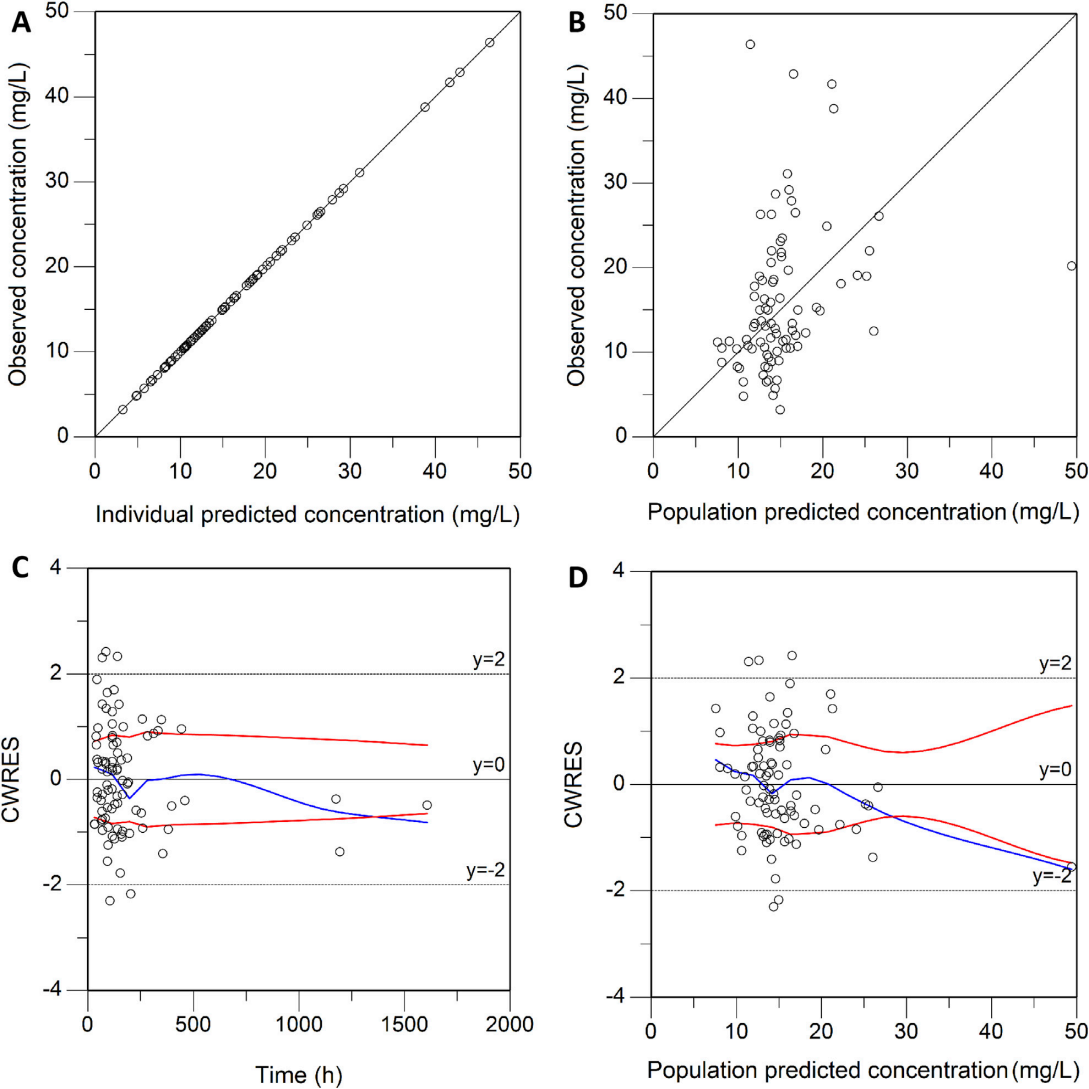
Goodness-of-fit plot of the final model. **(A)** Individual predicted concentration versus observed concentration. **(B)** Population predicted concentration versus observed concentration. **(C)** Conditional weighted residuals versus time. **(D)** Conditional weighted residuals versus population predicted concentration. The red lines in **(A,B)** represent the regression line, while the solid red lines in **(C,D)** indicate the position where conditional weighted residual equals 0.

**FIGURE 2:**
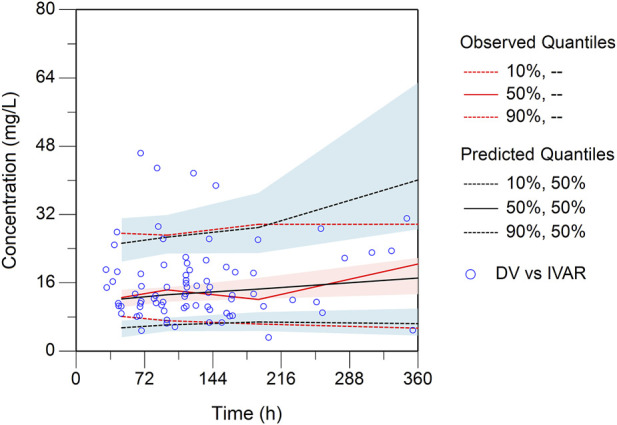
Plot of the prediction-corrected visual predictive check (n = 1,000). The blue dots are the measured concentrations. The red lines represent the 10%, 50%, and 90% percentiles of the observed concentrations. The blank lines represent the 10%, 50%, and 90% percentiles of predicted concentrations by the final model. The semitransparent shaded area represents the simulation-based 90% confidence interval (CI) for the corresponding predicted percentiles from the final model.

0.98 L/h and 108.69 L, respectively. These estimated PK parameters generally were in accord with the median values generated from the bootstrap method (CL, 0.97 L/h; V, 107.89 L) and fell within the 95% confidence intervals (CI) of the bootstrap results (Table 2), corroborating the stability of the model. The presence of CRRT significantly influenced the V of teicoplanin, such that V was 102.48% lower in the presence of CRRT (37.87L) than in the absence of CRRT (76.68L). Gender (males) was associated with a 1.90-fold higher V (31.85L with females versus 90.43L with males). The basic goodness-of-fit plots (Figure 1) demonstrated that the final model was appropriate, as both the predicted individual and population concentrations closely aligned with the observations. Moreover, the conditional weighted residuals were predominantly distributed randomly around the line of unity, within ±2 standard deviations, indicating the adequacy of the error model. The visual predictive check (VPC) results revealed that the 5th to 95th percentiles of the simulated data encompassed a majority of the observed data, further validating the model’s predictive accuracy (Figure 2).

**TABLE 2:** Population pharmacokinetic parameters of teicoplanin and bootstrap validation.

Parameters	Final model (n = 86)		Bootstrap (n = 1,000)	
	Estimate	RSE (%)	Median	95% CI
CL (L/h)	0.98	6.92	0.97	(0.83, 1.12)
V (L)	108.69	9.89	107.89	(84.30, 151.56)
θ_CRRT,V_	−0.71	22.55	−0.70	(-1.15, −0.19)
θ_Sex,V_	−1.07	28.53	−1.07	(-1.61, −0.25)
Between-subject variation				
ω^2^_CL_	0.31	17.58	0.31	(0.20, 0.41)
ω^2^_V_	0.09	32.11	0.08	(-0.05, 0.21)
Within-subject variation				
σ_additive_ (mg/L)	0.23	10.93	0.22	(0.03, 0.26)

### 3.3 Monte Carlo simulations and dosing optimization

Monte Carlo simulations (n = 1,000) were conducted using the final model to inform teicoplanin dosage selection. The virtual patients were stratified into three cohorts: male without CRRT (Group CRRT = 0), male receiving CRRT (Group CRRT = 1), and female without CRRT (Group Sex = 1). Only the AUCs/MIC and recommended regimens when MIC = 1 were simulated since the results were applicable to most scenarios with MIC≤1, and AUCs/MIC at other MIC levels can be extrapolated as needed.

**FIGURE 3:**
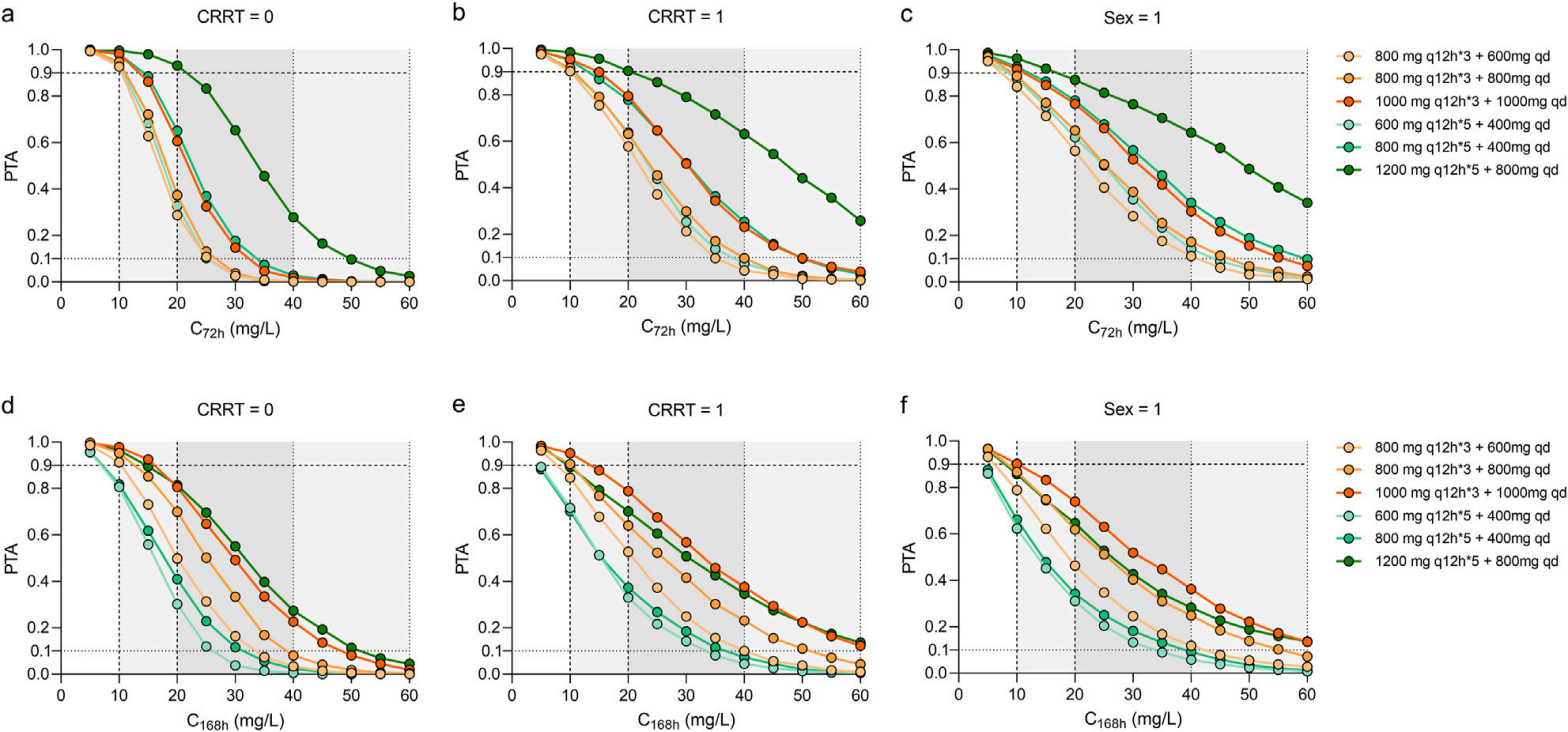
Monte Carlo simulations and probability of target attainment (PTA) for teicoplanin trough concentrations at 72 h (C_72_) and 168 h (C_168_), stratified by continuous renal replacement therapy (CRRT) status **(a,b,d,e)** and sex **(c,f)**. The teicoplanin dosage regimens were set at loading doses ranging from 600 mg to 1200 mg every 12 h for either 3 or 5 doses, with maintenance doses ranging from 200 mg to 1000 mg every day. Continue dosing regimens that administrated 400–1000 mg every 12 h.

**FIGURE 4:**
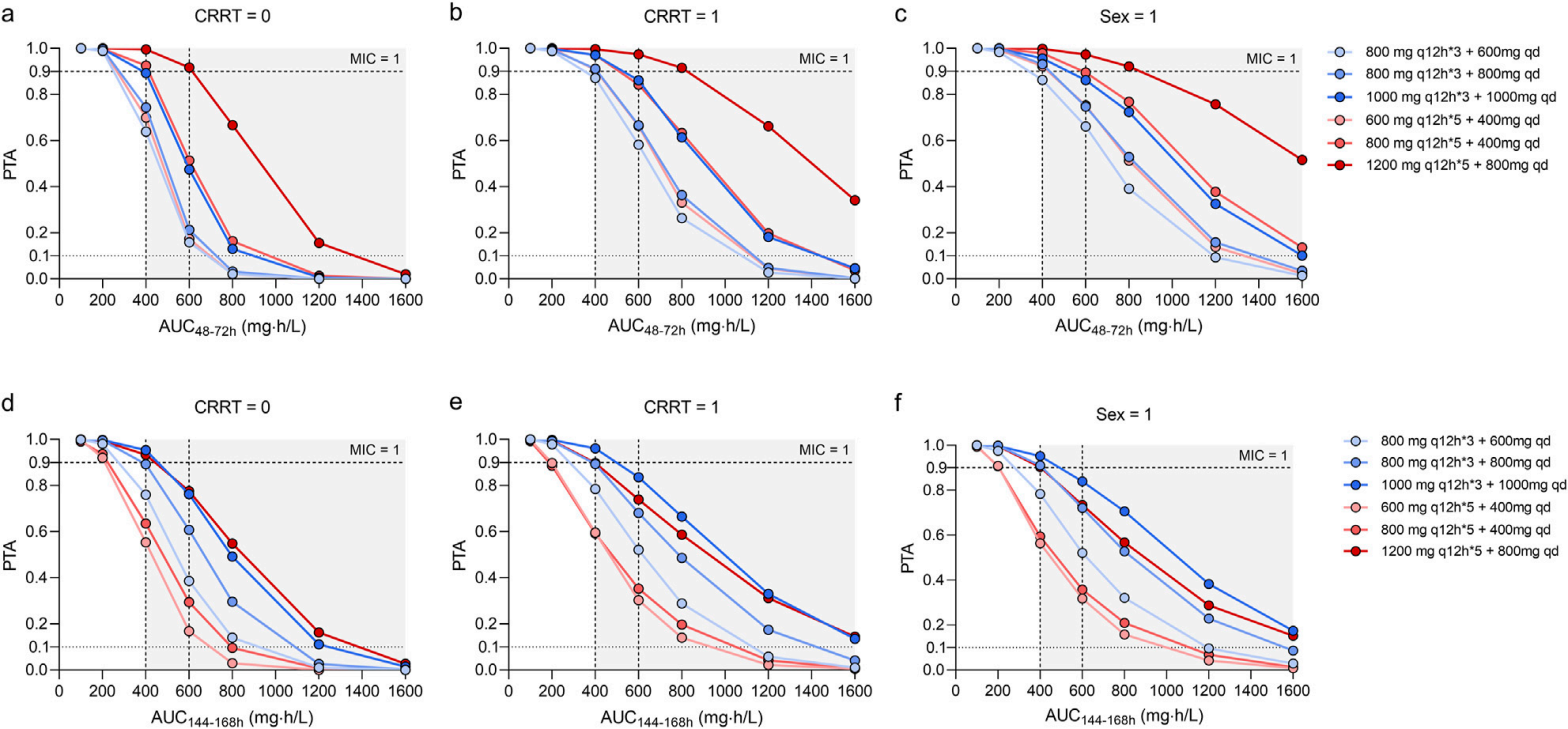
Monte Carlo simulations and probability of target attainment (PTA) for teicoplanin AUC_48-72h_ and C_144-168h_ values, stratified by continuous renal replacement therapy (CRRT) status **(a,b,d,e)** and sex **(c,f)**.

As shown in Figures 3, 4, the PTAs for steady state Cmin and AUCs of different teicoplanin dosing regimens were simulated across various CRRT statuses and genders. As expected, both teicoplanin Cmin and overall exposure generally increased with higher initial loading doses. Figure 4 showed the PTAs at the mean C_72h_ attained with various loading dose regimens. All simulated dose regimens were sufficient to achieve a mean C_72h_ of 10 mg/L in male sepsis patients with or without CRRT, with PTAs exceeding 90%. However, the dosage regimens (600 mg q12h*5 + 400 mg qd; 800 mg q12h*5 + 400 mg qd) had overall PTAs of 70%∼80% for C_168h_ ≥ 10 mg/L. As for female sepsis patients, an initial loading dose of 1,000 mg q12h for 3 days, followed by a maintenance dose of 1,000 mg q24h, was effective in achieving a mean C_72h_ of 10 mg/L. Only the regimen (1,000 mg q12h*3 + 1,000 mg qd) was effective in achieving a mean C_168h_ ≥ 10 mg/L, with an overall PTA of 90.20%. In all, fewer than 2% of sepsis patients exhibited potentially toxic concentrations (>60 mg/L) across the simulated dosing regimens, illustrating that all strategies evaluated in this study provided acceptable exposure levels.

As presented in Figure 4, the dosing regimens (1,200 mg q12h*5 + 800 mg qd; 600 mg q12h*5 + 400 mg qd; 1,000 mg q12h*3 + 1,000 mg qd) exhibited overall PTAs ranging from 86.70% to 100.00% for AUC_48–72h_/MIC and AUC_144–168h_/MIC ≥ 400. However, the regimen (1,200 mg q12h*5 + 800 mg qd) resulted in a AUC_48–72h_/MIC ≥1,600, potentially leading to toxic effects due to overexposure.

**FIGURE 5:**
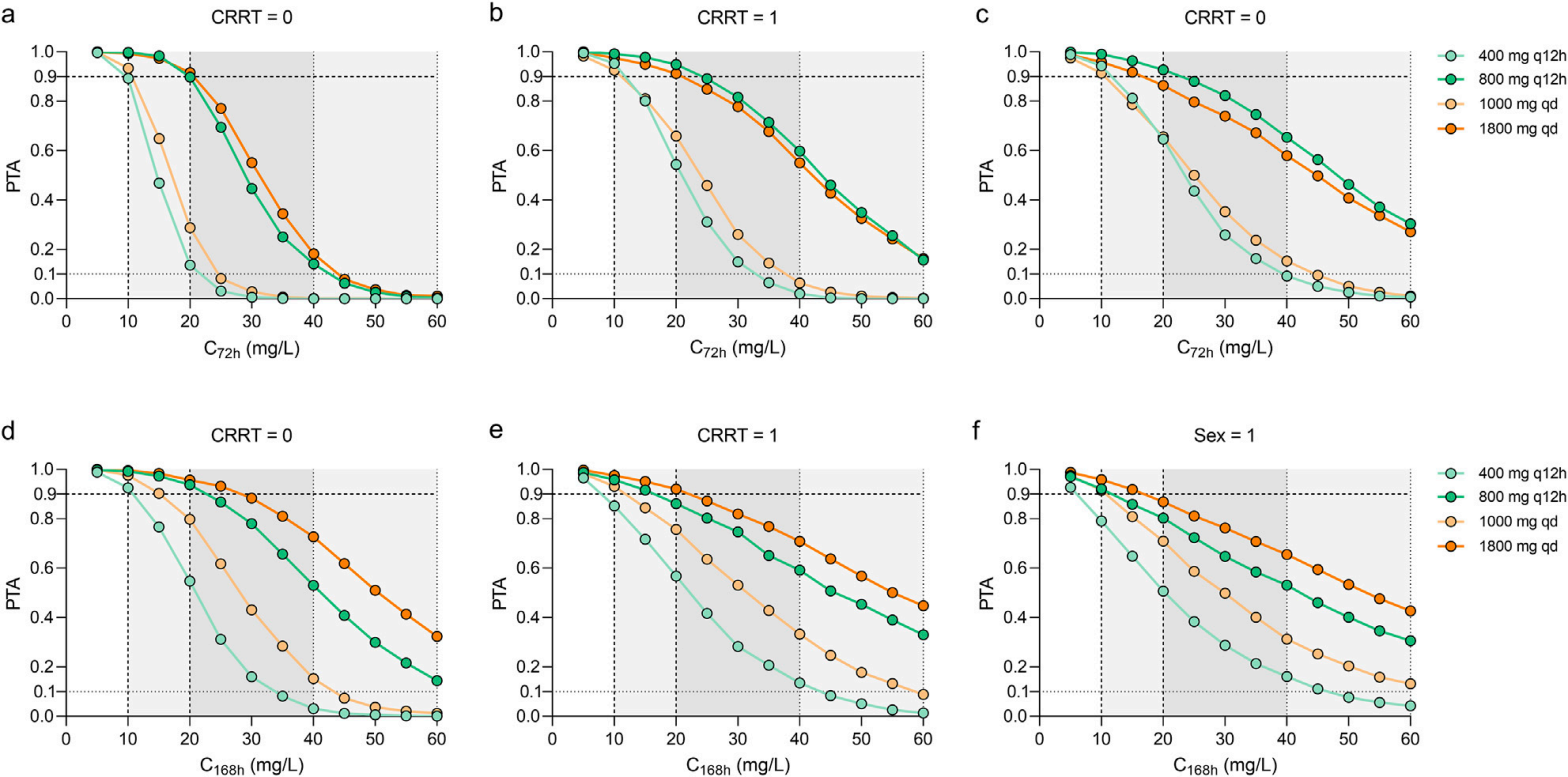
Monte Carlo simulations and probability of target attainment (PTA) for C_72h_ and C_168h_ values stratified by continuous renal replacement therapy (CRRT) status **(a,b,d,e)** or sex **(c,f)** for various dosing regimens. Continue dosing regimens that administrated 400 and 800 mg every 12 h or 1000–1800 mg every day were investigated.

At the specified maintenance doses (Figure 5), the concentration-time curves of 800 mg, q12h and 1800 mg, qd showed similar trends across the three groups of sepsis patients, while the dosing regimen of 1800 mg, qd achieved a potentially toxic exposure. At best, 1,000 mg per day and 400 mg, q12h at maintenance achieved PTAs ≥90% at a mean C_72h_ of 10 mg/L. Higher maintenance doses were required for sepsis patients with CRRT and female patients.

In conclusion, the findings suggested that optimal dosing regimens of teicoplanin for sepsis patients (MIC = 1) were as follows: sepsis patients (males) without CRRT need three loading doses of 800 mg q12h followed by a maintenance dose of 600 mg q24h; sepsis patients (males) with CRRT need three loading doses of 800 mg q12h followed by a maintenance dose of 800 mg q24h; female patients with sepsis require 1,000 mg q12h followed by a maintenance dose of 1,000 mg q24h.

## 4 Discussion

Teicoplanin is a glycopeptide antibiotic used to treat infections caused by methicillin-resistant *S. aureus* (MRSA), a major pathogen in sepsis. Due to its extensive application in critically ill patients recently and the considerable variability in homoeostatic changes and interventions, teicoplanin exhibits significant variability in pharmacokinetics. Considering the relative paucity of pharmacokinetic study and evidence-based dosing guidelines for teicoplanin in sepsis patients, especially those requiring special interventions such as CRRT or ECMO, a single-site clinical study was conducted. The study proposed optimal teicoplanin dosing regimens for sepsis patients undergoing CRRT based on population pharmacokinetics modeling and Monte Carlo simulations. The observed concentration-time data for teicoplanin were best depicted by a one-compartment model, consistent with a pharmacokinetic study in adult patients with sepsis (Fu et al., 2022). Goodness-of-fit plots, visual predictive checks and bootstrap results demonstrated the satisfactory performance of our final population PK model. Overall, teicoplanin clearance estimated in our current study was generally consistent with previously reported values (Kang et al., 2023; Ogawa et al., 2013; Chen et al., 2023), showing relatively prolonged CL (0.98 L/h vs. 0.38–1.03 L/h). This may be attributable to the characteristics of our study population, who were more seriously ill, older age [i.e., median (IQR): 62.0 (53.0, 71.2) years], had lower albumin levels [i.e., median (IQR): 31.6 (29.5, 39.4) g/L] and smaller body size. However, the volume of distribution were larger than the earlier estimates (Kang et al., 2023; Ogawa et al., 2013; Chen et al., 2023), with a slightly increased volume of distribution (V = 108.69 L). However, the volume of distribution was larger than earlier estimates (V = 108.69 L), suggesting a potential impact of clinical status or supportive therapies. In our cohort, early initiation of CRRT for fluid overload may have contributed to hemodynamic stabilization and reduced interstitial fluid accumulation, potentially lowering the volume of distribution (Pistolesi et al., 2019; Bugge, 2004). Teicoplanin’s high protein binding and naturally limited distribution, in combination with ultrafiltration or adsorptive filters, may have further restricted its tissue penetration. CRRT may also influence the free drug fraction by altering the protein-binding equilibrium, especially in hypoalbuminemic patients, and the type of CRRT membrane used (e.g., high-flux filters with adsorptive properties) may influence teicoplanin kinetics (Toutain and Bousquet-Mélou, 2004). Moreover, important CRRT-related variables—such as effluent flow rate, filter adsorption capacity, dialysis timing relative to dosing, and treatment modality (e.g., CVVH vs. CVVHDF)—were not fully captured in our retrospective dataset, thereby introducing residual variability not accounted for in the model.

In this study, covariate analysis revealed a significant correlation between CRRT and the volume of distribution of teicoplanin. Our finding was different to those of other studies, which typically reported an increased volume of distribution for drugs prescribed during CRRT. Few clinical studies have investigated teicoplanin pharmacokinetics in sepsis patients undergoing CRRT. Teicoplanin is predominantly eliminated by the kidneys, with its clearance typically dependent on renal function and CRRT (Kang et al., 2023; Ogawa et al., 2013; Chen et al., 2023). Notably, the inclusion of CRRT as a factor in clearance did not enhance model performance. Instead, volume overload--the primary reason for initiating CRRT-may have contributed to the reduced volume of distribution of teicoplanin. Previous studies failed to provide conclusive data on the impact of CRRT on teicoplanin PK, potentially due to patient baseline variability, differences in CRRT modality and intensity, and the lack of detection of nonrenal excretion of teicoplanin (Wi et al., 2017; Ueda et al., 2014). Additionally, the polymethylmethacrylate membrane used in continuous hemodiafiltration may be responsible for the adsorption of teicoplanin (Sakr et al., 2018). Hemodilution, altered protein binding, and other pathophysiologic changes occurring during CRRT may have a significant impact on the pharmacokinetics of teicoplanin (Expert group of antimicrobial dosing optimization during continuous renal replacement therapy, 2024). As most previous studies were conducted using *ex vivo* systems or in neonates or children, these findings may not be directly applicable to the adult patients in the present study (Goulenok and Fantin, 2013; Zhang et al., 2020). Moreover, gender-related difference has been recognized as a significant determinant for teicoplanin pharmacokinetics. An increased trend in the volume of distribution was observed in female sepsis patients in this study. The mechanisms underlying gender-specific pharmacokinetics can be attributed to both physiological and molecular factors, including reduced activity of drug-metabolizing enzymes, smaller organ size, lower body weight [median (IQR): males, 66.0 (54.0, 73.0) vs. females, 57.0 (47.0, 63.5)], a higher percentage of body fat, lower glomerular filtration rate, and different gastric motility in female compared with male (35 females and 51 males) (Meibohm et al., 2002; Strenger et al., 2013). Otherwise, gender differences in plasma protein levels, including albumin and α1-acid glycoprotein, may affect the protein binding of teicoplanin, which is highly protein-bound. Variations in protein binding can alter both the total and free drug concentrations, influencing drug distribution and pharmacodynamics (Lakhani et al., 2006). Sex-related hormonal differences and their effects on renal and hepatic physiology may also contribute to variability in drug pharmacokinetics, although teicoplanin is predominantly renally eliminated and minimally metabolized (Chow et al., 1993).

Similar to this current study, previous studies indicated that the currently approved dosages of teicoplanin may result in inadequate systemic exposures and suboptimal antibacterial activity for patients undergoing CRRT (Byrne et al., 2018). A population PK study involving five CRRT patients suggested that dose adjustment might not be necessary for those receiving renal replacement therapy (Wi et al., 2017). However, our study, which included a total of 20 CRRT patients, identified CRRT as a significant covariate influencing the volume of distribution, indicating that the pharmacokinetics of teicoplanin during CRRT treatment cannot be overlooked. Despite observing a reduced volume of distribution and no apparent changes in the teicoplanin clearance during CRRT, the PTAs was consistently lower in the presence of CRRT across all dosing regimens. This suggests substantial pharmacokinetic variability among patients, which is further reflected in the high relative standard error (RSE) associated with the interindividual variability in CL. When considering the final population PK model and its variability, our dosing simulations demonstrated a gradual increase in PTA from 72 to 168 h after the initiation of CRRT. Based on these findings, we recommend the following dosing regimens for patients with MIC≤1 mg/L: sepsis patients (males) without CRRT need three loading doses of 800 mg q12h followed by a maintenance dose of 600 mg q24h; sepsis patients (males) with CRRT need three loading doses of 800 mg q12h followed by a maintenance dose of 800 mg q24h. Our recommendations align with increasing evidence in the literature supporting the use of higher teicoplanin doses in critically ill patients (Ogawa et al., 2013; Pea et al., 2004; Hiraki et al., 2015). It is crucial to closely monitor teicoplanin plasma concentrations and the clinical status of patients to ensure effective infection control and minimize the risk of developing antimicrobial resistance.

This study has several limitations that should be acknowledged. First, the retrospective design inherently carries a risk of selection bias, incomplete data capture, and residual confounding. Although efforts were made to standardize data collection and minimize heterogeneity, the lack of prospective control limits causal inference between covariates and pharmacokinetic parameters. Second, as a single-center study conducted at a tertiary-care academic hospital, the generalizability of our findings may be limited. Institutional factors-such as CRRT modalities, antimicrobial stewardship practices, and therapeutic drug monitoring protocols—can vary significantly across different settings. These variations may affect teicoplanin pharmacokinetics and the clinical applicability of our dosing recommendations. Therefore, while our model provides a useful framework for dose individualization in sepsis patients, further validation in larger, multicenter prospective studies is warranted to confirm its external validity and to refine the dosing strategy under diverse clinical conditions. Third, only blood samples were analyzed to determine plasma concentrations, and the non-renal excretion of teicoplanin was not assessed. While plasma concentrations guide dosing, they may not reflect tissue drug levels, especially in critically ill patients with impaired perfusion. Teicoplanin shows limited distribution in sites like lungs and CSF. Non-renal clearance may also be underestimated due to prevalent renal dysfunction and CRRT in our cohort. Given the retrospective design and lack of tissue or excretory sampling, tissue-specific PK and non-renal pathways could not be evaluated. Future prospective studies with site-specific monitoring and full PK profiling are needed to refine dosing in patients with atypical drug distribution or clearance. Finally, while physiological changes associated with altered protein binding can significantly influence the pharmacokinetics of highly protein-bound drugs-particularly those with a low extraction ratio like teicoplanin-only total concentrations of teicoplanin were measured, with unbound concentrations not accounted for (Roberts et al., 2014; Wong et al., 2013). This limitation highlights the need for future studies incorporating free-drug measurements and albumin-adjusted pharmacokinetic modeling to improve individualized dosing. Accordingly, the proposed dosing regimens should be interpreted with caution, as they are based on total rather than unbound teicoplanin concentrations.

## 5 Conclusion

In conclusion, individualized dosing is essential to optimize teicoplanin therapy in sepsis patients undergoing CRRT. The effects of CRRT and gender on teicoplanin exposure should be acknowledged. A model-based teicoplanin dosing regimen for patients with CRRT was proposed, and the use of higher teicoplanin doses in critically ill patients is recommended; however, prospective validation of this is needed.

## Data Availability

The original contributions presented in the study are included in the article/supplementary material, further inquiries can be directed to the corresponding author.
